# Selenium-Loaded Calcium Phosphate with Long-Term and Curative Dosage Drug Release for Post-Surgical Osteosarcoma Management and Osteogenesis

**DOI:** 10.3390/ijms27146408

**Published:** 2026-07-18

**Authors:** Yang Yan, Yue Chen, Danjie Meng, Shidong Liu, Yuxin Wan, Zhenze Xie, Dong Xu, Chang Du

**Affiliations:** 1 Zhejiang Hospital, School of Medicine, Zhejiang University, Hangzhou 310006, China; yanyang@zuaa.zju.edu.cn; 2 Zhejiang Provincial Clinical Research Center for Oral Diseases, Stomatology Hospital, School of Medicine, Zhejiang University, Hangzhou 310006, China; chenyue@zdkq.com.cn; 3 Key Laboratory of Oral Biomedical Research of Zhejiang Province, Stomatology Hospital, School of Medicine, Zhejiang University, Hangzhou 310006, China; 4 Department of Pediatric Dentistry, Stomatology Hospital, School of Medicine, Zhejiang University, Hangzhou 310006, China; mengdanjie@zjkq.com.cn; 5 Department of Pathology, Stomatology Hospital, School of Medicine, Zhejiang University, Hangzhou 310006, China; 7624036@zjuss.cn; 6 Department of Biomedical Engineering, School of Materials Science and Engineering, South China University of Technology, Guangzhou 510641, China; wanyuxin0508@126.com (Y.W.); xiezhenze@scut.edu.cn (Z.X.); 7 National Engineering Research Center for Tissue Restoration and Reconstruction, South China University of Technology, Guangzhou 510006, China; 8 Key Laboratory of Biomedical Materials and Engineering of the Ministry of Education, Innovation Center for Tissue Restoration and Reconstruction, South China University of Technology, Guangzhou 510006, China

**Keywords:** osteosarcoma, calcium phosphate, selenite, controlled release, redox homeostasis, bone regeneration, antibacterial activity

## Abstract

Although neoadjuvant chemotherapy is widely used after osteosarcoma (OS) surgery, suboptimal chemotherapy accelerates OS recurrence, and postoperative bone repair remains challenging. Here, we report a selenium-loaded biomimetic calcium phosphate (Se@BioCaP) to provide a long-term therapeutic-dose release for postoperative OS treatment. Sodium selenite was incorporated into a biomimetic calcium phosphate (BioCaP) via a wet biomimetic mineralization protocol. The release kinetics of Se@BioCaP achieved a transition from non-Fickian transport to Fickian diffusion, delivering sustained cytotoxic concentrations under acidic, tumor-like conditions while maintaining biocompatibility with osteoblasts (OBs) under physiological conditions. The reduction in OS cell viability was associated with the disruption of redox homeostasis, including the downregulation of key antioxidant enzymes (SOD2 and GPx1), increased reactive oxygen species (ROS), and acidic vesicular organelle (AVO) formation. Notably, Se@BioCaP200 extracts maintained in vitro anticancer and pro-osteogenic activity at day 42, while antibacterial activity was maintained through day 42. These findings suggest that Se@BioCaP may be a promising candidate for postoperative OS management.

## 1. Introduction

Although neoadjuvant chemotherapy has been widely used after surgery to reduce OS recurrence [1], inadequate chemotherapy is associated with a lower 5-year survival [2]. Sublethal drug concentrations may stimulate OS proliferation through hormesis [3], a process closely linked to autophagy [4,5]. Meanwhile, abnormal vasculature in the OS tumor microenvironment (TME) can cause uneven drug distribution [6], leaving some regions exposed to sublethal concentrations that may contribute to OS recurrence. Chemotherapy also induces osteoblast (OB) apoptosis [7], and causes immunosuppression [8], which significantly increases the risk of opportunistic infections [9,10]. Opportunistic infections caused by Gram-negative bacteria such as *Escherichia coli* (*E. coli*) are common complications in 44% to 94% of patients with malignancies and are associated with mortality [11,12].

To address these clinical issues, many multifunctional biomaterials have been developed to achieve local drug delivery at postoperative OS sites. Bone substitutes, including calcium phosphate [13], bioglass [14], hydrogels [15], and hybrid coatings [16] loaded with therapeutic molecules, represent a widely adopted strategy to facilitate bone regeneration after OS resection [17,18,19].For example, Hu et al. developed a TME-responsive gold-platinum nanoparticles-incorporated injectable nanocomposite oxaliplatin hydrogel for chemo-photothermal and oxaliplatin-mediated immunotherapy [20], which showed excellent TME remodeling and localized synergistic cancer therapy. Wang et al. also functionalized a clinically commonly used bone substitute with cisplatin, showing a great balance between biosafety, anticancer efficacy, and osteogenic activity [21]. The OS recurrence, bone repair, and opportunistic infections can extend for several months [22,23,24,25], while most biomaterials, especially in the aforementioned studies, maintain adequate drug release only for a few hours or several days, which cannot fulfill clinical needs.

First-line anticancer drugs primarily act by inducing DNA damage. OS cells can evade this cytotoxicity by overexpressing multidrug resistance (MDR) proteins like P-glycoprotein to stimulate the DNA repair mechanisms [26]. In this context, Se has emerged as a promising agent due to its safety and multiple anticancer mechanisms [27]. Unlike classic drugs, Se acts as a pro-oxidant within the tumor’s redox-imbalanced environment, catalyzing the generation of excessive intracellular ROS [28]. Se-based systems have therefore been engineered for local postoperative OS management to avoid systemic toxicity and support bone regeneration. Constructing multifunctional biomaterials, such as mesoporous silica nanoparticles or nanotube arrays [29,30], could repair bone-resection defects and modulate localized redox. Recent studies have shown that stimuli-responsive nano-Se delivery systems, including pH- and redox-dual sensitive matrices and hierarchically constructed bone mimetic minerals, can enhance intracellular drug delivery, enable pH-/redox-responsive ion release, and amplify anti-OS efficacy [30,31]. This ROS-mediated efficacy is more difficult for cancer cells to resist compared to single-target agents. However, Se-incorporated mesoporous silica nanoparticles merely function as Se delivery carriers for OS suppression and lack inherent osteogenesis, making them incapable of simultaneously repairing bone defects after OS resection. Therefore, it is urgent to develop bone substitutes that can promote osteogenesis, function as scaffolds, and inhibit OS recurrence after tumor surgery. Among various carriers, BioCaP is an attractive candidate for postoperative OS treatment. It can load bone morphogenetic protein [32], vascular endothelial growth factor [33], strontium [34], etc., and has shown cell-mediated and responsive drug release [35]. Unlike other carriers such as metal–organic frameworks or hydrogels, BioCaP stands out due to its good biocompatibility, cell-mediated drug release, and inherent chemical similarity to natural bone tissue [36,37]. The nature of BioCaP is octacalcium phosphate (OCP) [38], which has a unique advantage due to its isomorphous substitution capability [39,40]. The BioCaP crystals allow for the co-precipitation of Se into the crystals to replace phosphate (PO_4_^3−^) groups. This atomic-level integration ensures a stable distribution and sustained release of Se, which is essential for modulating the interfacial interactions between the scaffold and the biological microenvironment.

However, functional bone repair in the postoperative TME remains a significant challenge due to the pathological disruption of bone homeostasis [41]. Maintaining the balance between bone resorption and formation is essential for the long-term success of surgery [42]. Within this regulatory framework, osteopontin (OPN), receptor activator of nuclear factor-kappa B ligand (RANKL), and osteocalcin (OCN) serve as critical parameters for evaluating the bone remodeling status and terminal mineralization [43]. Therefore, it is imperative to investigate whether the bone substitute can effectively modulate these key markers to restore the bone resorption balance and foster a favorable microenvironment for sustained bone repair.

**Figure 1:**
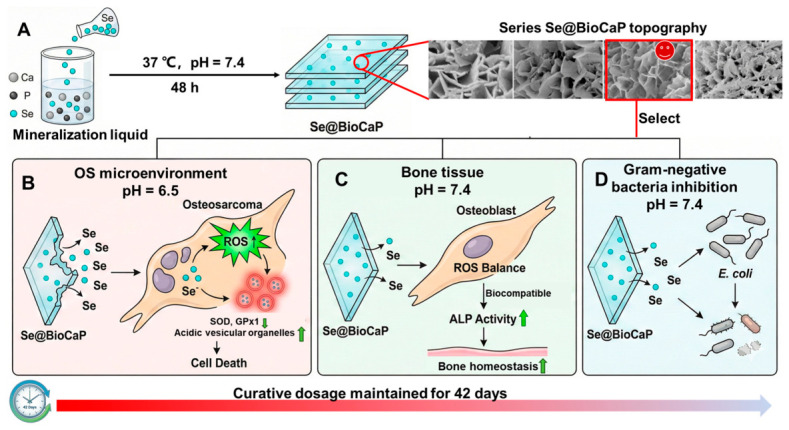
Schematic illustration of Se@BioCaP synthesis and its anticancer, osteogenesis, and antibacterial activities. (**A**) Se (0–1200 μg/mL) was incorporated into calcium phosphate crystals via wet biomimetic mineralization. The Se@BioCaP crystal size decreased as the Se concentration increased, and Se@BioCaP200 (was labeled with red smile face) selected for further assessment. (**B**) Proposed mechanism by which Se@BioCaP induces OS cell death through ROS-mediated acidic vesicular organelle (AVO) accumulation. (**C**) Se@BioCaP scaffolds exhibited biocompatibility with OB cells and upregulated ALP expression to promote bone repair. (**D**) Se@BioCaP achieved at least 8 weeks of sustained, pH-responsive drug release and induced long-term AVO stimulation.

In this study, Se@BioCaP was developed by wet biomimetic mineralization. The material provides pH-responsive therapeutic dose release for up to 42 days, thereby inhibiting OS cell viability through ROS-mediated AVO accumulation. This multifunctional strategy addresses the combined requirements of OS recurrence control and infection prevention and establishes a dosage-time synchronization approach for long-term post-operative OS treatment (Figure 1).

## 2. Results

### 2.1. Preparation and Morphological Characterization of Se@BioCaP

**Figure 2:**
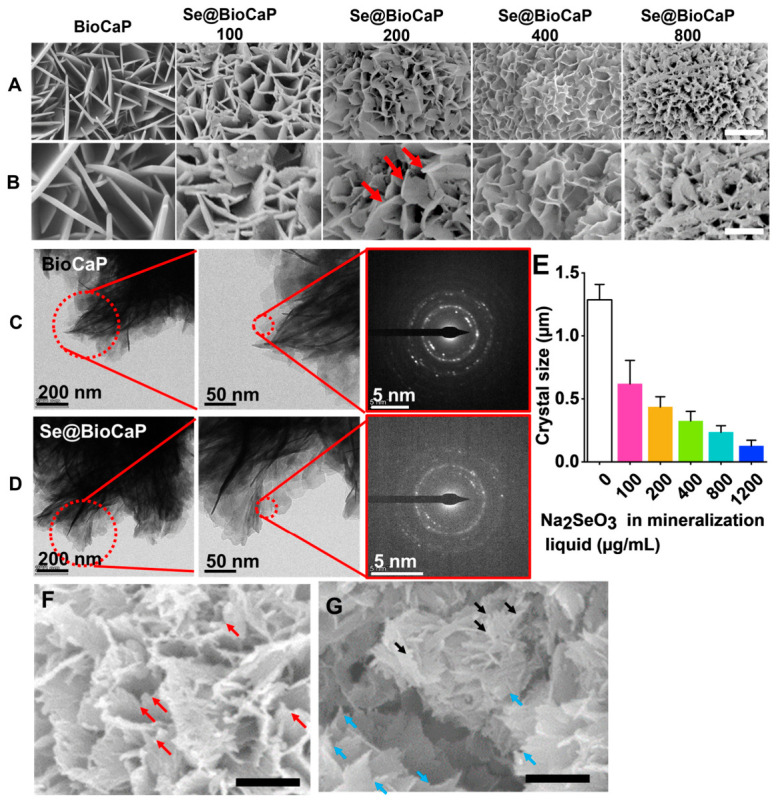
Composition and morphological characterization of BioCaP and Se@BioCaP. (**A**) SEM images of BioCaP and Se@BioCaP coatings prepared with increasing Na_2_SeO_3_ concentrations in the mineralization solution (0–1200 µg/mL), scale bar = 500 nm. (**B**) Higher magnification SEM images showing the crystal micro/nanostructure change from large straight BioCaP crystals to smaller, curved Se@BioCaP crystals/rods. Scale bar = 250 nm. (**C**,**D**) TEM images and SAED patterns confirming the crystal phases of the Se@BioCaP and BioCaP. (**E**) Quantitative analysis showing the dose-dependent decreased average crystal size. Data are presented as mean ± SD from three independent experiments (n = 3); (**F**) SEM image of Se@BioCaP crystal degradation in PBS after 14 days; crystal defects on the crystal edges are indicated by red arrows. Scale bar = 250 nm. (**G**) SEM images of Se@BioCaP crystal degradation in PBS after 28 days; extensive defects on the crystal edge are indicated by blue arrows and needle-like crystals by black arrows. Scale bar = 250 nm.

Inspired by biological mineralization processes, we fabricated a series of Na_2_SeO_3_ co-precipitated biomimetic calcium phosphate biomaterials by adding 100, 200, 400, 800, and 1200 µg/mL Na_2_SeO_3_ during crystallization. The resulting samples were named Se@BioCaP100, Se@BioCaP200, Se@BioCaP400, Se@BioCaP800, and Se@BioCaP1200, respectively. SEM analysis revealed that Se@BioCaP exhibited a greater crystal density than the Na_2_SeO_3_-free BioCaP crystals (Figure 2A). Se incorporation altered crystal morphology. Compared with the large, straight BioCaP crystals (Figure 2B), Se@BioCaP crystals were smaller and curved. In addition, Se also affected the crystal size. The average crystal size of BioCaP was 1.3 ± 0.1 µm, which significantly decreased to 0.6 ± 0.2 µm for the Se@BioCaP100 group. As the Na_2_SeO_3_ concentration of mineralization liquid increased to 800 µg/mL, the crystal size dose-dependently decreased from 1.3 ± 0.1 µm to 0.2 ± 0.05 µm (Figure 2E). SAED patterns (Figure 2C,D) showed typical diffraction spots for the (010) and (002) planes, which corresponded to octacalcium phosphate (OCP), confirming that both BioCaP and Se@BioCaP maintained the OCP crystal phase [44]. Thus, while Na_2_SeO_3_ tailored the crystal topography (i.e., shape, density, and size), it still preserved the intrinsic OCP crystal phase.

We also monitored the morphological changes in Se@BioCaP200 via SEM at 14 and 28 days to understand its long-term stability and degradation behavior. For the Se@BioCaP crystal at day 0, the crystal edges were intact, and the surface was free of impurities (Figure 2B, red arrows). At day 14, crystal defects on the crystal edges were observed (Figure 2F, red arrows), indicating initial crystal degradation. More pronounced crystal degradation and phase transformation were observed at day 28. The SEM revealed more extensive defects on the crystal edge (Figure 2G, blue arrows) and numerous needle-like crystals on the surfaces of the original crystal plates were also observed (Figure 2G, black arrows).

### 2.2. Se Loading and Release Kinetics of Se@BioCaP

**Figure 3:**
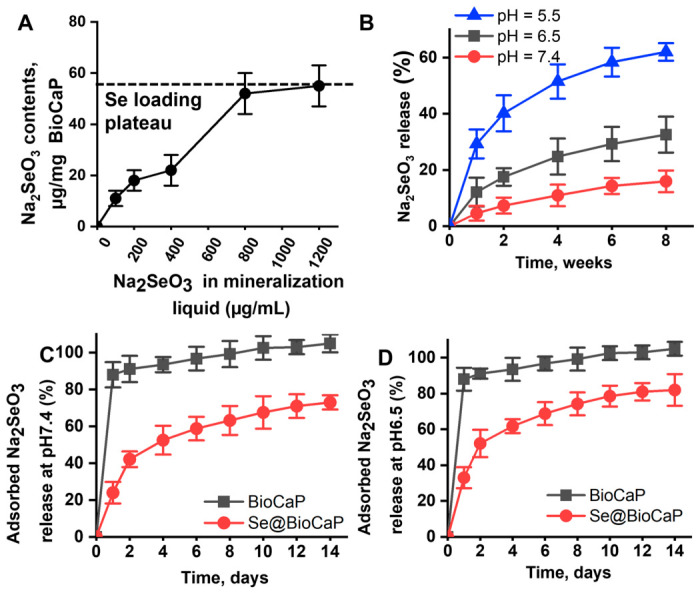
Drug loading and release from Se@BioCaP. (**A**) The Na_2_SeO_3_ loading content in various Se@BioCaP mineralized forms from different Na_2_SeO_3_ concentration solutions. (**B**) Cumulative Na_2_SeO_3_ release from Se@BioCaP under different simulated pH conditions over 8 weeks. (**C**) Cumulative Na_2_SeO_3_ release at pH 7.4 from Se@BioCaP or BioCaP with additional surface-absorbed Se. (**D**) Cumulative Na_2_SeO_3_ release at pH 6.5 from Se@BioCaP or BioCaP with additional surface-absorbed Se. Data are presented as mean ± SD from three independent experiments (n = 3).

An important aspect of the co-precipitation process is understanding its loading capacity. Figure 3A shows that Na_2_SeO_3_ incorporation into BioCaP was dose-dependent but reached a plateau. The maximum Na_2_SeO_3_ loading was 59 µg Na_2_SeO_3_ per mg BioCaP, with a plateau between 800 and 1200 µg/mL Na_2_SeO_3_ in the mineralization solution. The therapeutic efficacy of a local drug delivery system strongly relies on its release kinetics. Se was first adsorbed on the BioCaP or Se@BioCaP200 surfaces. BioCaP released almost 90% of Se within the first day, and Se@BioCaP showed a slower, sustained release than that of BioCaP. Although Se adsorbed onto Se@BioCaP showed sustained release at pH 6.5 and 7.4, burst release remained evident (Figure 3C,D), which could increase the risk of systematic selenite toxicity [45], such as peripheral neuropathy and digestive system stimulation [45,46]. In contrast, co-precipitated Se@BioCaP was engineered to provide a prolonged bioactive sustained release. Se@BioCaP achieved sustained release for at least 8 weeks (Figure 3B). Therefore, co-precipitation of Se in Se@BioCaP is a safe and effective drug delivery strategy, which is promising for locally preventing OS recurrence and promoting long-term osteogenesis. More importantly, the release from the optimized Se@BioCaP was highly pH-responsive and adaptive (Figure 3B). At pH 7.4, which mimics healthy bone conditions, Se@BioCaP released only 16 ± 4% of loaded Se by week 8. At pH 6.5, which mimics the OS microenvironment [47], release increased to 33 ± 6%, which allows for targeted drug action within the tumor microenvironment. The even faster release observed at pH 5.5, which mimics endosomal/lysosomal conditions during cellular internalization [48], reached 62 ± 3% by week 8.

**Figure 4:**
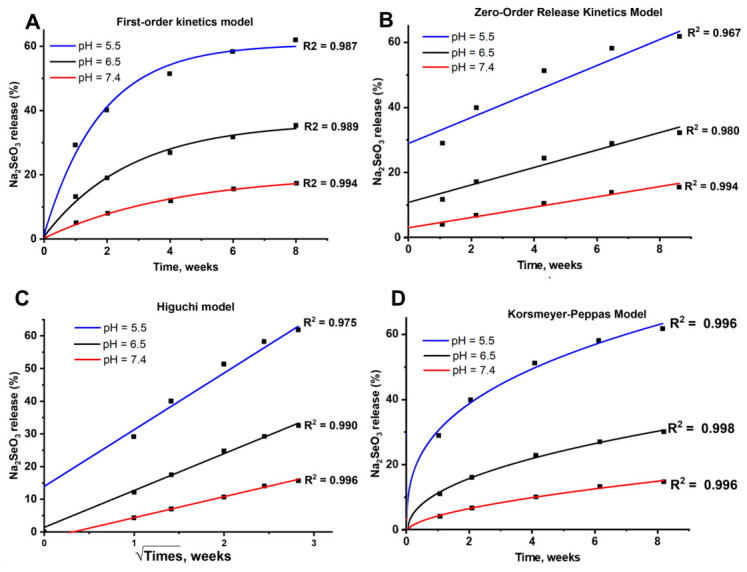
Release-kinetics fitting of Se@BioCaP at pH = 5.5, 6.5, and 7.4. (**A**) First-order release kinetics; (**B**) Zero-order kinetics model; (**C**) Higuchi model; (**D**) Korsmeyer–Peppas model.

To quantitatively define the Na_2_SeO_3_ release behavior from the Se@BioCaP under physiological and TME-mimicking pH conditions, the release kinetics were fitted using four classical kinetic models: zero-order release kinetics, the first-order kinetics model, the Higuchi model, and the Korsmeyer–Peppas model. The fitting curves and corresponding adjusted R-square (R^2^) are presented in Figure 4. A higher R^2^ indicates a suitable model for describing the release kinetics [49]. Both the first-order kinetics model and the Korsmeyer–Peppas model fit well with the Se release profiles across all tested pH levels. Notably, R^2^ for the Korsmeyer–Peppas model were above 0.995 for pH 7.4, 6.5, and 5.5, which were higher than the zero-order kinetics, Higuchi, and first-order models. The Korsmeyer–Peppas model provided the highest R^2^ value, closest to 1.0, indicating that it is the most accurate model for predicting the release mechanism from the Se@BioCaP crystals.

The release exponent (n) in the Korsmeyer–Peppas equation can be used to predict the specific nature of drug release [49]. For a plate-like or flake-like matrix, an n value below 0.45 indicates Fickian diffusion, whereas 0.45 < n < 0.89 indicates non-Fickian transport, in which release is governed by diffusion, matrix erosion, or transformation [50]. The fitting results for Se@BioCaP showed a significant pH-dependent transition in the release mechanism (Figure 4D). In a physiological environment (pH 7.4), the n value is 0.583, falling within the range of 0.45 to 0.89 (Table 1), suggesting non-Fickian transport [51]. We speculate that OCP slowly transforms into the more stable HA phase. This mechanism ensures sustained stability and safety of the Se@BioCaP in healthy bone tissue. In the TME-mimicking condition (pH 6.5), the n value shifted to 0.460, which is very close to Fickian diffusion (0.45), indicating that as the environment becomes slightly acidic, the structural constraints of the crystalline lattice show a much higher degradation potential [51]. In lysosomal or highly acidic environment (pH 5.5), the n value further decreased to 0.346 (n < 0.45), indicating that the release pattern transitions into a typical Fickian diffusion mechanism [52]. In the zero-order kinetics model, the fitted slope (K_0_) characterizes the pH-responsive release rate of Se@BioCaP. Table 1 shows that under OS-mimicking acidic conditions (pH 6.5), the release rate slope is 0.0288. In contrast, under physiological conditions (pH 7.4), the slope decreases to 0.01701. Calculations reveal that the release rate at pH 6.5 is approximately 1.7 times higher than that at pH 7.4, confirming the accelerated Se release in acidic environments.

### 2.3. Anticancer Activity and Its Mechanisms of Se@BioCaP in OS Cells

**Figure 5:**
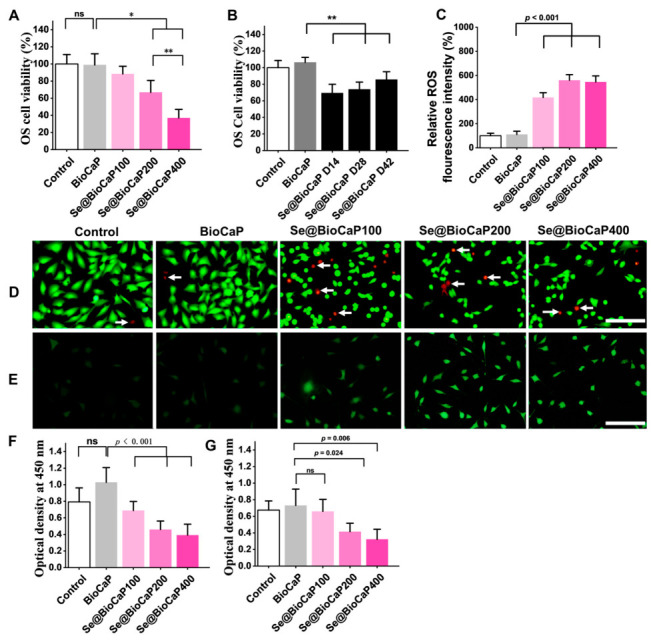
Long-term inhibition of OS cell viability, ROS induction, and endogenous antioxidant enzyme activity (SOD2 and GPx1). (**A**) Viability of human 143B OS cells after 2 days of treatment with BioCaP or Se@BioCaP extracts (n = 3). (**B**) Viability of OS cells after 2 days of treatment with BioCaP or Se@BioCaP200 extracts collected after 14, 28, and 42 days of soaking (n = 3). (**C**) Quantification of ROS fluorescence intensity in OS cells after 1 day of treatment with BioCaP or Se@BioCaP extracts (n = 3). (**D**) Live/Dead staining of 143B OS cells after treatment with Se@BioCaP; white arrows indicate dead OS cells. Scale bar = 200 µm. (**E**) ROS staining of Se@BioCaP and BioCaP-treated OS cells at day 1. Scale bar = 200 µm. (**F**) SOD2 activity in OS cells after treatment with BioCaP or Se@BioCaP extracts for 1 day (n = 3). (**G**) GPx1 activity in OS cells after 1 day of treatment with BioCaP or Se@BioCaP (n = 3). Data are presented as mean ± SD from three independent experiments (n = 3); One-way analysis of variance (ANOVA) with Bonferroni’s post hoc test was used to test differences between groups; ns, not significant; * *p* < 0.05; ** *p* < 0.01.

The anticancer activity of Se@BioCaP was quantified after 2 days of treatment. Se@BioCaP200 and Se@BioCaP400 reduced OS cell viability to 66.76% ± 14% and 36.87% ± 10% respectively (Figure 5A). The long-term anticancer efficacy of the Se@BioCaP was evaluated by treating 143B OS cells with Se@BioCaP200 conditioned media collected at 14, 28, and 42 days (Figure 5B). BioCaP did not show any OS inhibition effects; the 14-day extract of Se@BioCaP200 inhibited OS cell viability to 69.39% after 2-day treatment, which was the greatest OS viability inhibition among all groups (Figure 5B). These results demonstrated a potent, dose-dependent inhibition. This sustained effect was also evidenced by the 28-day and 42-day Se@BioCaP200 extracts. Live/Dead staining showed a significant decrease in cell density and changes in cell morphology after treatment with the diverse Se@BioCaP extracts (Figure 5D, Scale bar = 200 µm) for 1 day. The surviving OS cells in Se@BioCaP100, Se@BioCaP200, and Se@BioCaP400 groups showed a significantly decreased cell density, and most OS cells became shrunken, which is consistent with cell death (Figure 5D). This potent anticancer activity was linked to the induction of severe oxidative stress, as evidenced by a 4- to 6-fold intracellular ROS increase in the Se@BioCaP100, Se@BioCaP200, and Se@BioCaP400 groups (Figure 5C,E).

To evaluate the intracellular redox response, the levels of key antioxidant enzymes, SOD2 and GPx1, were quantified through ELISA (Figure 5F,G). The BioCaP group showed an increase in SOD2 (1.03 ± 0.18) and no elevation in GPx1 (0.73 ± 0.20) compared to the control group (0.79 ± 0.17 and 0.68 ± 0.11, respectively), indicating that the calcium phosphate matrix itself maintains or slightly stimulates the cellular antioxidant defenses. However, Se@BioCaP triggered a significant, dose-dependent downregulation of both enzymes. Se@BioCaP200 significantly inhibited SOD2 to 0.46 ± 0.10, while GPx1 decreased to 0.41 ± 0.10. Se@BioCaP400 showed the most pronounced inhibitory effect, with SOD2 and GPx1 levels further declining to 0.39 ± 0.13 and 0.32 ± 0.12, respectively. These quantitative results confirm that Se@BioCaP effectively compromises the enzymatic antioxidant defense system in OS cells.

**Figure 6:**
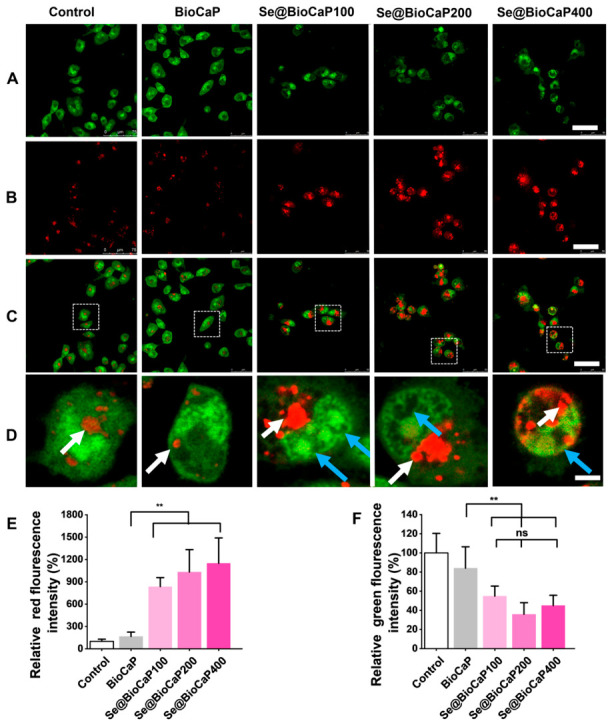
Detection of AVO in Se@BioCaP-treated OS cells by AO staining and LCSM. (**A**) Green fluorescence showing AO-stained cytoplasm and nuclei in 143B OS cells after treatment with BioCaP or Se@BioCaP extracts. Scale bar = 50 μm. (**B**) Red fluorescence showing AVO in 143B OS cells. Scale bar = 50 μm. (**C**) Merged green and red fluorescence images. Scale bar = 50 μm. (**D**) High-magnification images from dashed box of (**C**) showing AVO (white arrows) and nuclear fragmentation (blue arrows). Scale bar = 5 μm. (**E**,**F**) Quantification of mean red and green fluorescence intensity. Red fluorescence indicates AVO accumulation; green fluorescence indicates cytoplasm/nuclei. Data are presented as mean ± SD from three independent experiments (n = 3); ANOVA with Bonferroni’s post hoc test was used to test differences between groups; compared with BioCaP, ns, not significant; ** *p* < 0.01.

Confocal microscopy observations of AO-stained OS cells provided further insight into the OS cell death. Green fluorescence represents the stained cytoplasm and nucleus, while the AVOs associated with autolysosome formation were stained with red fluorescence. The cell morphology showed an elongated spindle shape after treatment with complete growth medium or BioCaP (Figure 6A), and no obvious AVOs were observed (Figure 6D), indicating no cytotoxicity to OS cells. Critically, bright red fluorescent puncta were densely aggregated in the cytoplasm in the Se@BioCaP100, Se@BioCaP200, and Se@BioCaP400 groups (Figure 6D), and their fluorescence intensity was 8–10 times higher than that of the control group (Figure 6E), indicating significantly induced AVO accumulation. The dramatic accumulation of red fluorescent AVOs in the Se@BioCaP groups represents the OS cells’ attempt to degrade and recycle damaged components (Figure 6D). Green fluorescence in the Se@BioCaP100, Se@BioCaP200, and Se@BioCaP400 groups significantly decreased to 55%, 35, and 44% when compared to the control group, respectively (Figure 6F).

### 2.4. Osteogenic Differentiation and Bone Homeostasis of Se@BioCaP

**Figure 7:**
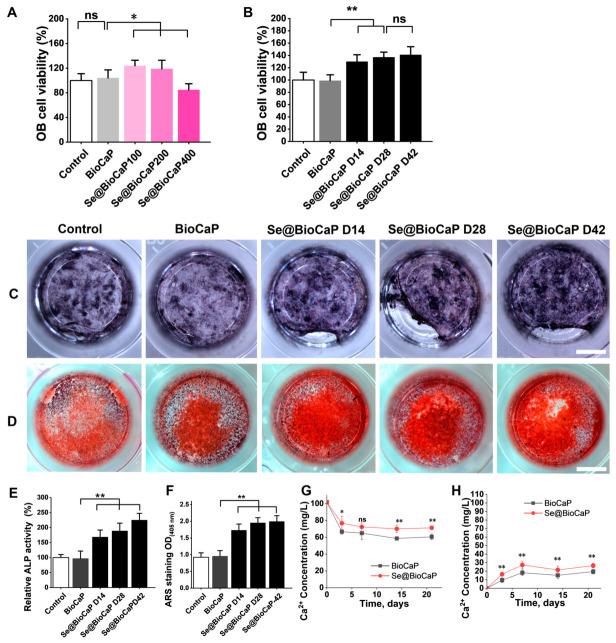
Biocompatibility of BioCaP and Se@BioCaP and osteogenic differentiation capacity of Se@BioCaP200 in OB cells. (**A**) Viability of OB cells after 2 days of treatment with Se@BioCaP or different BioCaP samples. (**B**) Viability of OB cells after treatment with BioCaP or Se@BioCaP200 extracts collected after day 14, day 28, or day 42 of soaking. (**C**) Representative BCIP/NBT staining images showing ALP activity after 14 days of osteogenic induction. Scale bar = 2000 µm. (**D**) Representative ARS images showing mineralized nodules after 21 days of osteogenic induction. Scale bar = 2000 µm. (**E**) Quantification of ALP activity after treatment with BioCaP or Se@BioCaP200 extracts collected after day 14, day 28, and day 42 of soaking. (**F**) Quantification of matrix mineralization nodules by ARS after treatment with BioCaP or Se@BioCaP200 extracts collected after day 14, day 28, and day 42 of soaking. (**G**) Ca^2+^ concentrations in SBF after soaking BioCaP or Se@BioCaP200 for up to 21 days. (**H**) Ca^2+^ concentrations in calcium-free saline after soaking BioCaP or Se@BioCaP200 for up to 21 days. Data are presented as mean ± SD from three independent experiments (n = 3); ANOVA with Bonferroni’s post hoc test was used to test differences between groups; ns, not significant; * *p* < 0.05; ** *p* < 0.01.

The biocompatibility and osteogenic activity of Se@BioCaP and BioCaP were evaluated in MC3T3-E1 pre-osteoblasts. BioCaP, Se@BioCaP100, and Se@BioCaP200 demonstrated good biocompatibility. Se@BioCaP100 and Se@BioCaP200 significantly increased OB cell viability to 123.8% ± 9% and 118.8% ± 14%, respectively, after 2 days of culture (Figure 7A). In contrast, Se@BioCaP400 decreased the OB cell viability to 84.7% ± 10%, indicating that low-Se loading groups (Se@BioCaP100 and Se@BioCaP200) showed better biocompatibility with OB cells. Therefore, long-term biocompatibility assays were carried out for the Se@BioCaP200 group and showed significant OB cell viability when treated with 14-, 28-, and 42-day extracts of Se@BioCaP200 (Figure 7B). The key finding was enhanced osteogenesis compared to the control group. Se-free BioCaP did not significantly promote the expression of the early osteogenic marker ALP. Se@BioCaP D14, Se@BioCaP D28, and Se@BioCaP D42 significantly upregulated ALP activity by 1.8-, 2.0-, and 2.2-fold when compared to the control group, and there were no significant differences among the Se@BioCaP D14, D28, and D42 groups (Figure 7C,E). The late-stage osteogenic effect of Se@BioCaP200 was further confirmed by matrix mineralization nodule formation; Se@BioCaP200 extracts from different time points significantly increased ARS by 1.73- to 1.99-fold compared to the control group, whereas BioCaP did not significantly increase the ARS (Figure 7F). The qualitative results also confirmed the pro-osteogenic effect. As visualized by BCIP/NBT staining, OB cells treated with Se@BioCaPs showed a deeper purple color, indicating higher ALP activity compared to the control groups. Subsequently, Se@BioCaP showed the formation of extensive, densely stained red calcified nodules compared to the BioCaP groups (Figure 7B,C).

The Ca^2+^ ion exchange behavior of BioCaP and Se@BioCaP200 was further evaluated. In SBF (initial Ca^2+^ concentration: 101.69 mg/L), the BioCaP group rapidly consumed Ca^2+^, with the Ca^2+^ concentration decreasing from 101.69 mg/L to 64.96 mg/L by day 7. In contrast, the Se@BioCaP group showed a smaller reduction in Ca^2+^ concentration and maintained higher Ca^2+^ levels than the BioCaP group throughout the 21-day observation period (Figure 7G). In calcium-free saline, the Se@BioCaP200 group exhibited a significantly faster Ca^2+^ release compared to the BioCaP group (Figure 7H), indicating that Se doping effectively promoted the degradation of BioCaP crystals. This accelerated degradation may be related to the higher crystal density and thus larger specific surface area (Figure 2A), which could facilitate ion exchange and release.

### 2.5. Antibacterial Activity of Diverse Se@BioCaP

**Figure 8:**
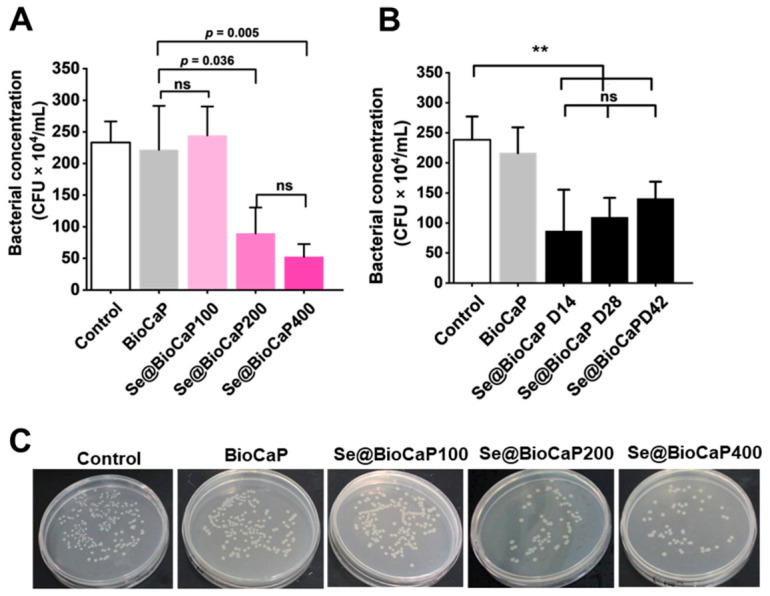
*E. coli* concentrations in a broth inoculation assay of diverse Se@BioCaP and BioCaP. (**A**) *E. coli* counts after 18 h of treatment with BioCaP or different Se@BioCaP extracts (n = 3). (**B**) *E. coli* counts after treatment with Se@BioCaP200 extracts collected after 14, 28, or 42 days of soaking (n = 3). (**C**) Representative colony-counting images after 18 h of treatment. Data are presented as mean ± SD from three independent experiments (n = 3); ns, not significant. ANOVA with Bonferroni’s post hoc test was used to test differences between groups; ns, not significant; ** *p* < 0.01.

Se@BioCaP and BioCaP (200 mg) were soaked in 100 mL of growth medium at 37 °C to obtain the supernatant for evaluating their antibacterial effects. Diverse Se@BioCaP and BioCaP extracts were used to assess antibacterial activity against *E. coli* and select an effective antibacterial formulation (Figure 8). The Control, BioCaP, and Se@BioCaP100 groups showed similar mean CFU counts, indicating no detectable antibacterial activity. After 18 h of treatment, the Se@BioCaP200 and Se@BioCaP400 groups significantly reduced the *E. coli* CFU to 86.3 × 10^4^ and 52.7 × 10^4^ CFU/mL, respectively, compared with the BioCaP group (Figure 8A,C). Long-term antibacterial activity was then assessed using Se@BioCaP200 extracts collected at days 14, 28, and 42 (Figure 8B). The Se@BioCaP200 extracts at day 14 and day 28 showed a significantly lower CFU when compared to the BioCaP group. However, no significant difference was observed between the Se@BioCaP D42 group and the BioCaP group.

Taken together, these findings demonstrate that Se@BioCaP is a biocompatible bone substitute, achieves an environment-adaptive release transition from non-Fickian transport to Fickian diffusion, delivering a sustained OS lethal dosage at acidic tumor pH while ensuring biosafety under physiological conditions. The released Se downregulated SOD2 and GPx1, promoted ROS accumulation, and increased AVO formation. Se@BioCaP also supported osteogenic differentiation and provided sustained antibacterial activity up to day 28, although antibacterial activity was not significant on day 42.

## 3. Discussion

Although advanced multifunctional biomaterials using photothermal, sonodynamic, or immunotherapeutic approaches have demonstrated promising tumor eradication effects [53,54], such externally stimulated approaches often generate transient therapeutic effects. Few studies have verified whether sustained ion release can maintain therapeutic levels over clinically relevant durations because most biological tests focus on the initial burst-release phase [55,56]. Chemotherapy also causes immunosuppression, which induces opportunistic infections and compromises bone repair capacity [57,58,59]. Wang et al. loaded cisplatin into commercially available bone substitute granules for postoperative OS application [21]. Hu et al. combined chemotherapy, photothermal effects, chemodynamic therapy, and immunotherapy for localized synergistic cancer therapy [20]. Se has also been incorporated into HA and has shown good anticancer and osteogenesis efficiencies, but with 60% of the Se released on the first day [31]. Therefore, a co-precipitation strategy for the long-term release of Se from BioCaP was proposed in this study, with Se selected as a prototypical therapeutic molecule.

In this research, Se was co-precipitated into BioCaP crystals and was found to regulate crystal growth. Se@BioCaP exhibited a significantly greater crystal density than the Na_2_SeO_3_-free BioCaP crystals (Figure 2A), which might be attributed to the effect of Na_2_SeO_3_ on the CaP phase composition of the obtained powders [60]. Se@BioCaP showed a decrease in crystal size as the Se concentration increased in the mineralization solution (Figure 2E). A robust Se@BioCaP loading increase between 400 and 800 μg/mL was observed, which is speculated to be driven by a synergistic transition in crystallization kinetics. Ma et al. showed similar evidence that Se is a potent crystal growth inhibitor for HA [61]. In this work, Se reduced the crystal size from the micrometer to the nanometer scale while driving morphological transition from straight plates to smaller curved plates and rods (Figure 2B,E). This dimensional collapse significantly increased the specific surface area, exposing abundant active binding sites that accelerated Se co-precipitation. However, approaching 1200 μg/mL, the loading curve reached a plateau; we speculated that available coordination sites may have reached saturation. Se@BioCaP exhibited an OCP phase (Figure 2C,D), which is widely regarded as a transient precursor phase to the more thermodynamically stable HA [62]. After 4 weeks of soaking, needle-like crystals were observed on the Se@BioCaP (Figure 2G, black arrow indicated). This observation may indicate a phase transfer from the initial OCP to the thermodynamically more stable HA [62], which is the principal inorganic constituent of natural bone [63]. Petrakova et al. showed a similar conclusion: OCP was soaked in DMEM [64], and they also observed a smaller crystal size with sharp points on the crystal edges at 28 days, with a considerable amount of HA and a small amount of OCP present after 44 days of soaking [65]. Such phase transformation consists of intense OCP dissolution [66], which was mostly driven by ion exchange in the initial weeks [67], with HA formation gradually occurring during long-term hydrolysis [65].

OCP crystals could incorporate or adsorb drugs (Figure 3C,D), however, a fundamental limitation of physical adsorption is rapid burst release, which results from weak surface interactions [68,69], e.g., Van der Waals forces and hydrophobic interactions [70,71]. This is unstable and non-responsive in a complex in vivo environment [70]. Although the topography of Se@BioCaP400 slightly tempered this burst after 2 days (Figure 3C,D), it is due to surface pores entrapping some of the drug [72], the release remained passive and diffusion-driven. However, the co-precipitation strategy achieves a true, sustained release by atomically integrating Se into the BioCaP crystal lattice. This ensures that drug release is intrinsically governed by the carrier’s own slow, cell-mediated degradation and ion exchange. To select the optimal kinetic model, two parameters were evaluated: the proximity of the R^2^ to 1.0, and the minimization of the residual sum of squares (RSS), which serves as a robust indicator of the model’s prediction error (Table 1). Among the four classical models tested, the Korsmeyer–Peppas model showed superior fitting performance across the three pH conditions (5.5, 6.5, and 7.4). Specifically, it consistently exhibited the lowest RSS values (all < 10^−3^) compared to the zero-order and Higuchi models, indicating that the experimental data deviated minimally from the model predictions. The pH-dependent shift in release dynamics (expressed in n values of the Korsmeyer–Peppas model) also illustrates the pH-responsive behavior of the Se@BioCaP system (Table 1). At physiological pH, Se ions are structurally integrated and locked in the OCP crystal lattice. However, in an acidic environment, the BioCaP crystals significantly degraded and dissolved, as evidenced by the n value and the formation of edge defects in SEM observations (Figure 2F,G). This acid-induced degradation unlocks the incorporated Se ions, allowing them to diffuse out rapidly through the porous matrix. This mechanism allows for a triggered burst of Se in the TME to maximize OS cell inhibition via ROS-mediated AVO formation while minimizing off-target toxicity in healthy bone tissues.

The substantially decreased SOD2 and GPx1 content (Figure 5F,G) provides a crucial link between ROS stimulation and the activation of AVOs. In this study, the observed defensive collapse, characterized by the sharp decline in these primary antioxidant enzymes, is interpreted as a prerequisite for autophagy. SOD2 and GPx1 are major antioxidant defenses against oxidative stress [73]; their exhaustion at high Se@BioCaP concentrations (200–400 µg/mL) leads to an irrecoverable accumulation of intracellular ROS. Kwatra et al. linked redox imbalance to autophagic dysregulation, demonstrating that weakened antioxidant defenses allow accumulated ROS to trigger stress-signaling pathways, such as ASK1, and lead to autophagy [74]. ROS-dependent endoplasmic reticulum stress and autophagy have been reported as core anti-tumor mechanisms [75]. Furthermore, the localized calcium ion release from the BioCaP matrix may synergistically enhance this effect by promoting mitochondrial membrane permeability [76], which exacerbates ROS leakage and accelerates the autophagic destruction of malignant cells [77]. Together, these effects suggest that Se@BioCaP disrupts redox homeostasis in OS cells. While these potent efficacies are highly desirable at the OS site, clinical translation of Se@BioCaP requires validation of its safety profiles. In patients with platinum-sensitive recurrent cancer, intravenous administration of high-dose Se (2000 µg/day) was reported to reduce motor neuropathy, particularly in patients aged 60 or older, without affecting long-term progression or cancer-specific survival. [78]. Crucially, while inorganic selenite could induce acute ROS-driven risks and organic compounds cause cumulative systemic retention [31,79], utilizing multifunctional biomimetic scaffolds enables controlled, cell-mediated Se release to avoid off-target damage to healthy tissue while preserving the pro-oxidant activity that activates caspase-dependent apoptotic cascades in residual OS cells [29,31].

According to a phase I randomized, double-blind controlled trial, daily oral supplementation with 400 µg sodium selenite, Se-methylselenocysteine (MSC), or seleno-L-methionine (SLM) for 8 weeks in cancer patients did not induce significant Se-related toxicities or severe treatment-emergent adverse events [80]. Furthermore, qPCR-based DNA damage quantification showed negligible genotoxicity in both nuclear DNA (nDNA) and mitochondrial DNA (mtDNA) in patient peripheral blood mononuclear cells at this 400 µg/day dose, and even suggested the potential to enhance normal cell DNA repair pathways [79]. However, distinct chemical forms introduce substantial variations in potential toxicity and accumulation that must be considered in biomaterial design. Whereas inorganic sodium selenite exhibits rapid plasma clearance in vivo, organic SLM is non-specifically incorporated into albumin, significantly elevating the plasma concentration and maintaining levels 50% above baseline even after a 4-week washout [80].Long-term exposure still poses latent risks that restrict the clinical translation of Se-based orthopedic implants [81]. Daily Se intake at 300 μg over five years was associated with elevated mortality after a decade, owing to disrupted selenoprotein equilibrium across visceral organs [81]. Adverse responses to chronic Se overload, such as alopecia, nail lesions, and chronic inflammatory lesions, have also been reported [82]. As the safety of long-term local accumulation of Se in patients with tumors remains unknown, Se@BioCaP also poses both translational challenges and critical biosafety concerns for clinical application.

While autophagy can initially serve as a cytoprotective mechanism, facilitating long-term tumor survival and recurrence within the complex TME [83], the persistent oxidative stress and the simultaneous degradation of antioxidant proteins through autophagic flux are likely to shift this process towards a lethal autophagic program [84]. Li et al. also suggest that autophagy could be a protective mechanism that degrades damaged organelles in quiescent OS celsl [85], which may promote OS cell survival [86] or metastasis when subjected to metabolic stress [87].However, Se@BioCaP released an adequate Se dose, as it not only increased red fluorescence from AO-stained AVOs (Figure 6C), but also showed significantly reduced green fluorescence, indicating widespread degradation of nucleic acids (DNA/RNA) [88]. The observation of hallmark morphological changes in OS cells, such as chromatin condensation, nuclear fragmentation, and a smaller, rounded morphology, provides strong evidence for apoptosis [89]. Unlike conventional chemotherapeutics such as doxorubicin or methotrexate, whose cytotoxic effects are often evaded by OS cells through upregulating MDR proteins and stimulating DNA repair mechanisms [90,91], Se is a pro-oxidant with multi-targeted anti-cancer activity. Se may act through multi-target oxidative stress pathways to initiate parallel signaling cascades, including p53 activation and Bcl-2/Bax modulation [92,93]. This study provides proof-of-principle evidence for the biological activity of Se@BioCaP in relation to OS inhibition, bone repair, ROS generation, AVO aggregation, and overall cell viability. Further research on specific markers for autophagy, such as LC3-II/Beclin-1 [94], or apoptotic cascades involving Caspase-3, will be carried out [95] to understand the crosstalk between autophagy and apoptosis in post-operative OS treatment.

A limitation of the study is that while the AO red-stained AVOs are a functional indicator of cellular acidification and autophagy induction [96,97], a comprehensive evaluation of the key proteins of autophagic initiation and execution was not performed; therefore, future investigations will focus on quantifying the dynamic expression of hallmark proteins, such as LC3-I/LC3-II and Beclin-1 [98], to fully elucidate the underlying molecular cascades. We also acknowledge that all assessments were performed in vitro. 2D cultured cells and in vitro release models cannot replicate the heterogeneous cellular ecosystem and bone microenvironment inside living organisms [99,100]. Follow-up in vivo experiments will verify the long-term OS, osteogenic and antibacterial effects of Se@BioCaP. Another limitation is the long-term accumulation, as the existing work mainly focuses on anticancer and osteogenic differentiation, which did not test the long-term immune response. The balance between persistent local Se accumulation and body fluid flow washout is still unknown; a long-term high local Se concentration may trigger chronic inflammation, which may raise immune biosafety concerns during clinical translation. Apart from in vivo biosafety concerns, the Se@BioCaP also faces manufacturing challenges that hinder standardized industrial production. Batch-to-batch variation mainly originates from unstable standard deviations of crystal size (Figure 2E) and drug loading (Figure 3A). Homogeneous Se distribution is a critical factor determining biosafety and therapeutic efficacy, thus process validation is still important before clinical translation.

Various Se-based biomaterials have been reported for OS therapy, while the present study differs in delivery performance and multifunctionality. Vundela et al. reported phytofabricated Se nanospheres, which showed promising biocompatibility, antimicrobial, and anticancer activities [101]. Meanwhile, Hu et al. also fabricated a Se-doped TiO_2_ nanotube array, which exhibited a rapid burst Se leakage to suppress OS growth [29]. However, the liquid–solid-solution of Se-doped TiO_2_ nanotube array synthetic involves multi-step liquid phase reactions and high-temperature hydrothermal treatment, which requires strict control over numerous experimental parameters. Such complicated fabrication procedures are highly sensitive to operating conditions and difficult to scale up for large-scale preparation. Mg, Fe, and Se nanocomposites achieve anti-tumor and osteogenic effects, but most ions were released within one week, mismatching long bone healing cycles [102]. In contrast, BioCaP is fabricated via a single-step procedure under physiological conditions (37 °C, pH 7.4), without the need for high temperatures, high pressures or repeated reagent addition steps, which is easy to scale up. Meanwhile, the current Se@BioCaP also enables pH-responsive Se release up to 42 days to achieve OS suppression, osteogenic differentiation, and antibacterial capacity simultaneously, addressing the major drawbacks of short duration and limited functionality in existing Se biomaterials.

The design of Se@BioCaP, as a proof-of-principle anticancer bone substitute, is to inhibit tumor cells while promoting osteogenesis. However, Se@BioCaP400 exhibited cytotoxicity to OBs (Figure 7A). Therefore, the primary focus was directed toward dosages lower than Se@BioCaP400, which could ensure an optimal therapeutic window that balances robust anticancer efficacy with biocompatibility. Future investigations will attempt to engineer structured coatings or add new therapeutic molecules to expand the therapeutic window of higher loading without compromising biocompatibility and osteogenesis. The OB cell viability of both BioCaP and Se@BioCaP groups exceeded 100% of the control level, indicating the biocompatibility of Se@BioCaP with OB cells. Baba et al. also demonstrated using an in vivo rat tibia model that OCP can activate OBs and promote bone regeneration even under osteoporotic conditions [103]. The SAED patterns of Se@BioCaP in our study (Figure 2C,D) confirmed that Se incorporation did not change the crystal phase of BioCaP. The Se@BioCaP maintained the intrinsic OCP crystal phase, which is a thermodynamically metastable crystal and tends to transform into hydroxyapatite (HA) under physiological conditions [104]. This phase transformation process typically involves the substantial consumption of exogenous Ca^2+^ from the surrounding environment [105]. However, our experimental results revealed that the reduction in Ca^2+^ concentration in the SBF medium was less pronounced for Se@BioCaP than for pure BioCaP (Figure 7G). These results showed a sophisticated dynamic competition between degradation-driven release and mineralization-driven adsorption at the OCP crystal interface. Due to the lattice distortion and significantly decreased crystal size of Se@BioCaP, the composite possesses larger specific surface area and chemical reactivity. Its enhanced degradation effectively compensates for the Ca^2+^ consumption during the mineralization process, thereby maintaining a higher level of localized Ca^2+^ concentration at the interface. This Se-induced localized hypercalcemic microenvironment holds profound clinical significance for bone regeneration following osteosarcoma resection. Consequently, the advantages of Se@BioCaP stem not only from the intrinsic biochemical activity of Se but also from its optimized regulation of Ca^2+^ kinetics. This dual ion-drug regulatory mechanism provides a highly efficient, bioadaptive strategy for the comprehensive management of postoperative osteosarcoma, addressing the critical needs of preventing recurrence, inhibiting infection, and promoting functional bone repair.

In summary, the key function of Se@BioCaP is to maintain a therapeutic and bioadaptive release over an extended period. Beyond sustained drug delivery, Se@BioCaP also modulates the local ionic microenvironment by regulating Se and Ca^2+^ fluxes and downregulating the antioxidant defense system (e.g., SOD2 and GPx1). This enzymatic depletion, together with the persistent release of Se and Ca^2+^, triggers ROS-mediated cell death. Meanwhile, the Se@BioCaP maintains sustained efficacy for at least six weeks, supporting anticancer activity and bone repair while also providing antibacterial activity during the release period. This multifaceted therapeutic strategy may offer a promising approach for the comprehensive and bioadaptive postoperative management of OS.

## 4. Materials and Methods

### 4.1. Materials

Simulated body fluid (SBF) was purchased from Sigma-Aldrich (St. Louis, MO, USA). Trypsin, Dulbecco’s Modified Eagle Medium (DMEM), and fetal bovine serum (FBS) were purchased from Gibco BRL (Gaithersburg, MD, USA). The Reactive Oxygen Species Assay Kit, Acridine Orange (AO) staining Kit, and Alamar Blue assay kit were purchased from Solarbio (Shanghai, China). Alizarin Red staining kit and Alkaline phosphatase assay kit were obtained from Beyotime (Shanghai, China).

### 4.2. Preparation of Se@BioCaP

A well-established wet biomimetic mineralization protocol was adopted [106]. Briefly, an accelerated biomimetic mineralization solution containing 684 mM NaCl, 13.5 mM KCl, 9 mM CaCl_2_·2H_2_O, 2.1 mM Na_2_HPO_4_·2H_2_O, 59.5 mM NaHCO_3_, and 5 mM MgCl_2_·6H_2_O (Sigma-Aldrich, St. Louis, MO, USA) was prepared at pH 6.0 and incubated at 37 °C for 24 h to form a thin amorphous CaP layer on the substrate. A supersaturated CaP solution containing 2 mM Na_2_HPO_4_·2H_2_O, 137 mM NaCl, and 4 mM CaCl_2_·_2_H_2_O was then prepared and buffered with 72 mM TRIS at pH 7.4. Na_2_SeO_3_ was added to this mineralization solution at 100, 200, 400, 800, or 1200 μg/mL. After 48 h, the resulting samples were designated Se@BioCaP100, Se@BioCaP200, Se@BioCaP400, Se@BioCaP800, and Se@BioCaP1200, respectively. For comparison, Se was physically adsorbed onto the surfaces of BioCaP or Se@BioCaP200 by soaking the samples in a 1200 μg/mL Na_2_SeO_3_ solution, followed by overnight lyophilization.

### 4.3. Composition Characterization of Se@BioCaP and BioCaP

The surface morphology and composition of the Se@BioCaP samples were characterized. A field-emission scanning electron microscope was performed using a, Nova Nano SEM 430 instrument (Thermo Fisher Scientific, Hillsboro, OR, USA) to observe the Se@BioCaP, BioCaP, or degraded Se@BioCaP morphology. Before SEM, BioCaP and Se@BioCaP samples were mounted on copper sheets and sputter-coated with a thin platinum layer. For microstructural and crystal phase analysis, transmission electron microscopy (TEM) images and selected area electron diffraction (SAED) patterns of BioCaP and Se@BioCaP were acquired using a Talos 120c instrument (FEI, Thermo Fisher Scientific, Hillsboro, OR, USA) operated at an accelerating voltage of 100 kV. The crystal length of BioCaP or Se@BioCaP was quantified via direct image analysis of SEM micrographs using ImageJ software. Six randomly selected visual fields from three independent batches were analyzed using ImageJ. To evaluate the Ca^2+^ ion exchange kinetics, BioCaP and Se@BioCaP200 were immersed in either calcium-free saline or simulated body fluid (SBF) incubated at 37 °C. At designated time points (up to 21 days), the soaking solutions were collected and replenished with fresh medium. The concentration of calcium ions in the collected supernatants was quantitatively analyzed using inductively coupled plasma optical emission spectrometry (ICP-OES, iCAP 7000, Thermo Fisher Scientific, Hillsboro, OR, USA). All experiments were performed in triplicate.

Se loading was quantified following acid digestion of the Se@BioCaP particles. Specifically, a 20 mg sample of Se@BioCaP was dissolved in 1 M HCl for 10 min. The supernatant was centrifuged for 5 min at 5000 rpm, and was isolated for analysis. The Se concentration was quantified by ICP-OES. The release kinetics were studied by incubating Se@BioCaP in phosphate-buffered saline (PBS) at 37 °C. Three separate repeated experiments were conducted using PBS buffered to pH 7.4, pH 6.5, and pH 5.5. At scheduled time points, the release medium was collected and refreshed with pre-warmed PBS. The supernatants were centrifuged at 5000 rpm for 5 min and were analyzed. The concentrations of released Se were quantified using ICP-OES. After 14 and 28 days of soaking, sample morphology was examined by SEM.

### 4.4. In Vitro Cell Viability Assessments

The bioactivity of the Se@BioCaP was tested on both OS (143B) and healthy pre-osteoblastic (MC3T3-E1) cell lines, purchased from ATCC (Manassas, VA, USA). These cells were cultured in DMEM supplemented with 10% fetal bovine serum (FBS) and 1% Penicillin-Streptomycin-Fungizone (Gibco, Gaithersburg, MD, USA) in a 37 °C, 5% CO_2_ environment. Various Se@BioCaP and BioCaP samples (200 mg) were soaked in a 100 mL growth medium at 37 °C to obtain the supernatant for evaluating their effects; the supernatant was collected every week. After screening, Se@BioCaP200 was selected. The supernatant of Se@BioCaP200 collected from day 8 to day 14 was named as Se@BioCaP D14; that collected from day 21 to day 28 was designated as Se@BioCaP D28. Supernatants collected at subsequent intervals were named accordingly. The cell viability (metabolic activity) was quantified using the Alamar Blue assay after the Se@BioCaP treatment. Specifically, cells were incubated for 4 h (under light-protected conditions) in 10% Alamar Blue working solution, and fluorescence was measured at 590 nm after excitation at 560 nm.

### 4.5. Oxidative Stress Assessment

143B OS or MC3T3E1 OB cells were seeded into 96-well plates at 1 × 10^4^ cells/cm^2^ density and incubated overnight for cell attachment. Next, they were subjected to a 24 h treatment with either the various Se@BioCaP extracts or complete growth medium, which served as a negative control. Following this treatment, the culture medium was discarded, and the cells were stained with a 5 µM solution of 2′,7′-dichlorodihydrofluorescein diacetate (DCFH-DA) in serum-free medium for 30 min at 37 °C in the dark. Finally, after carefully rinsing the cells to remove excess probe, the intracellular ROS fluorescence was imaged on a Leica fluorescence microscope (Leica Microsystems GmbH, Wetzlar, Germany) using an excitation of 488 nm and an emission of 520 nm. The average fluorescence intensity was then quantified by ImageJ software (NIH, Bethesda, MD, USA) to determine relative ROS levels.

### 4.6. ELISA Superoxide Dismutase (SOD2)

To quantify intracellular superoxide dismutase 2 (SOD2) levels, 143B OS cells were harvested, lysed, and subjected to analysis using an ELISA kit (Fine Test, Wuhan Fine Biotech Co., Ltd., Wuhan, China) in accordance with the manufacturer’s protocol. Briefly, the pre-coated 96-well plate (Greiner Bio-One GmbH, Frickenhausen, Germany) was rinsed twice. Subsequently, 100 μL of standards, controls, or OS cell lysate samples were added to wells, and incubated for 1.5 h at 37 °C. After removal of the liquid, the wells were washed twice, and 100 μL of biotin-labeled SOD2 antibody working solution was added, and the plate was incubated for another 1 h at 37 °C. Following aspiration and a three-wash cycle, the samples were incubated for 30 min at 37 °C with 100 μL of streptavidin-biotin complex (SABC) working solution. After 5 thorough washes, color development was initiated by introducing 90 μL TMB substrate for 15–30 min at 37 °C in the dark. 50 μL stop solution was added, and the optical density (OD) at 450 nm was immediately measured using a microplate reader (Synergy HTX, BioTek Instruments, Winooski, VT, USA).

### 4.7. ELISA Glutathione Peroxidase (GPx1)

To quantify intracellular GPx1, 143B OS cells were harvested, lysed, and subjected to analysis using an ELISA kit (Fine Test, Wuhan, China) in accordance with the manufacturer’s protocol. Briefly, the pre-coated 96-well plate was rinsed twice. Subsequently, 100 μL of standards, controls, or OS cell lysate samples were added to wells and incubated for 1.5 h at 37 °C. After removal of the liquid, the wells were washed twice, and 100 μL of biotin-labeled GPx1 antibody working solution was added. The plate was incubated for another 1 h at 37 °C. Following an aspiration and three-wash cycle, the samples were incubated for 30 min incubation at 37 °C. Following aspiration and three washes, the samples were then incubated with 100 μL of streptavidin-biotin complex (SABC) working solution for 30 min at 37 °C. After 5 thorough washes, color development was initiated by introducing 90 μL TMB substrate for 15–30 min at 37 °C in the dark. 50 μL stop solution was added, and the optical density (OD) at 450 nm was immediately measured using a microplate reader.

### 4.8. Acidic Vesicular Organelles (AVO) Analysis

The formation of AVO, a functional hallmark of the late-stage autophagic flux (i.e., autolysosome formation) [107], was assessed by acridine orange (AO) staining. Briefly, 2 × 10^4^ cells/cm^2^ OS cells were seeded onto a 24-well plate overnight. Following treatment with the respective BioCaP or Se@BioCaP conditioned media for 24 h, the cells were stained using an AO staining kit (Beyotime, Biotech Inc., Shanghai, China) for 15 min in a 37 °C incubator, with the plates protected from light. The cells were then washed carefully. The fluorescence was observed using a laser scanning confocal microscope (Leica Microsystems GmbH, Wetzlar, Germany). Green fluorescence (emitted by AO intercalated with nuclear DNA and cytoplasmic RNA) was detected using an excitation wavelength of 488 nm and an emission of 520 nm. Red fluorescence (emitted by AO aggregated in acidic compartments) was detected using the same 488 nm excitation laser but with a longer emission of 640 nm. The mean fluorescence intensity of green and red fluorescence was quantified by ImageJ software (v. 1.54j, NIH, Bethesda, MD, USA).

### 4.9. Osteogenesis Assay

The osteogenic differentiation potential of MC3T3-E1 cells was evaluated by monitoring alkaline phosphatase (ALP) activity and matrix mineralization nodules. OB cells were cultured in an osteogenic induction medium (standard growth medium supplemented with 10 mM β-glycerophosphate, 100 nM dexamethasone, and 0.2 mM ascorbic acid). This induction medium was refreshed every 2–3 days throughout the experiment. Early osteogenic activity was determined on day 14 by quantifying ALP levels using an ALP/BCA assay kit (Beyotime, Biotech Inc., Shanghai, China). Mineralized nodule formation was assessed by Alizarin Red S staining (ARS) on day 21. For ARS quantification, the red stain was eluted by cetylpyridinium chloride solution for 2 h at room temperature. The absorbance was measured at 405 nm by a microplate reader.

### 4.10. Antibacterial Assays

The antibacterial activity of Se@BioCaP was evaluated against the Gram-negative bacteria (*E. coli*). *E. coli*. was cultured in 2 mL Luria–Bertani broth at 37 °C for 24 h with 5% CO_2_, until the bacterial density reached 1.0 × 10^8^−1.0 × 10^9^ CFU/mL (CFU: colony forming unit). The bacterial suspension was serially diluted, and the OD_600_ value was measured using a multifunctional microplate reader to calculate the bacterial concentration. Next, the bacterial solution was adjusted to 10^6^ CFU/mL. Se@BioCaP. PBS extract (900 μL) was mixed with 100 μL of bacterial suspension in 1.5 mL tubes. Then, the mixtures were serially diluted 10-fold (100 μL of mixture + 900 μL of PBS buffer), vortexed, and 10 μL aliquots were spread onto agar plates. After incubation at 37 °C for 18 h, colonies were counted.

### 4.11. Statistical Analysis

All quantitative data are expressed as the mean ± standard deviation (SD). Statistical analyses were performed by SPSS software (Version 24.0, IBM, New York, NY, USA). Three independent experiments were performed, with three replicate wells per experiment. An independent sample *t*-test was utilized to compare two groups; one-way analysis of variance (ANOVA) followed by Bonferroni post hoc correction was applied for multiple group comparisons. For all statistical tests, a *p*-value of less than 0.05 was statistically significant.

## 5. Conclusions

Se-mediated crystallization tailored the micro-/nanostructure of BioCaP and enabled a pH-responsive Se release. Se@BioCaP released sustained cytotoxic Se concentrations to suppress OS while maintaining biocompatibility with OBs. Unlike conventional multifunctional biomaterials that offer only short-lived efficacy, Se@BioCaP extracts collected on day 42 retained in vitro anticancer capacity, highlighting their long-term potential. Se@BioCaP extracts induced the depletion of the antioxidant defense system, as indicated by SOD2 and GPx1 downregulation. The subsequent ROS increase drove significant AVO accumulation, a hallmark of late-stage autophagic flux, while Se@BioCaP also showed antibacterial activity against opportunistic pathogens such as *E. coli*. Meanwhile, Se@BioCaP modulated the local ionic microenvironment by regulating Se and Ca^2+^ fluxes, providing biochemical cues to support bone repair. Overall, this multifunctional therapeutic strategy may offer a promising approach for comprehensive and bioadaptive postoperative OS management.

## Figures and Tables

**Table 1:** Drug release kinetics fitting of Se@BioCaP under different pH conditions.

pH	The Release Kinetics Models		
	Zero-order release kinetics		
	Equation	R^2^	Residual sum of squares
5.5	y = 0.292 + 0.042 × x	0.967	2.296
6.5	y = 0.11246 + 0.0288 × x	0.980	0.375
7.4	y = 0.03504 + 0.01701 × x	0.988	0.2433
First-order release model			
	Equation	R^2^	Residual sum of squares
5.5	y = 0.606 − 0.596 × e^1.79963 × x^	0.987	0.00213
6.5	y = 0.333 − 0.323 × e^2.7559 × x^	0.989	0.000472
7.4	y = 0.186 − 0.1844 × e^4.24014 × x^	0.994	0.000063
Korsmeyer–Peppas model (y < 60%)			
	Equation	R^2^	Residual sum of squares
5.5	y = 0.310 × x^0.3459^	0.996	0.000884
6.5	y = 0.1270 × x^0.4608^	0.998	0.000120
7.4	y = 0.0486 × x^0.5831^	0.996	0.0000484
Higuchi model			
	Equation	R^2^	Residual sum of squares
5.5	y = 0.173 × x^1/2^ + 0.140	0.975	0.711
6.5	y = 0.113 × x^1/2^ + 0.0141	0.990	0.045
7.4	y = 0.0643 × x^1/2^ − 0.0179	0.996	0.0315

**Table 1.** Mathematical modeling and fitting for the in vitro release kinetics of Se from Se@BioCaP under acidic and physiological pH environments (pH 5.5, 6.5, and 7.4). Four classic kinetic models, including Zero-order, First-order, Korsmeyer–Peppas, and Higuchi models, were applied to describe the dissolution and diffusion mechanisms. The corresponding fitting equations, correlation coefficients (R^2^), and residual sum of squares are summarized.

## Data Availability

The data presented in this study are openly available in [Mendeley] at [https://doi.org/10.17632/b5gfv5g6nt.2].

## Abbreviations

The following abbreviations are used in this manuscript:

OS	Osteosarcoma
HA	Hydroxyapatite
Se	Selenium
BioCaP	Biomimetic calcium phosphate
Na_2_SeO_3_	Sodium selenite
Se@BioCaP	Selenium-loaded biomimetic calcium phosphate
OB	Osteoblast cells
MDR	Multidrug resistance
RANKL	Receptor activator of nuclear factor kappa-B ligand
OCN	Osteocalcin
OPN	Osteopontin
SOD2	Superoxide dismutase 2
GPx1	Glutathione peroxidase 1
SBF	Simulated body fluid
DMEM	Dulbecco’s modified Eagle medium
FBS	Fetal bovine serum
AO	Acridine orange
TEM	Transmission electron microscopy
SAED	Selected area electron diffraction
PBS	Phosphate-buffered saline
ALP	Alkaline phosphatase
ARS	Alizarin Red S staining
AVO	Acidic vesicular organelles
